# Study of Dielectric and Thermal Conductivity Properties of Recyclable Epoxy Resin Reinforced with SiO_2_-Coated Poly(p-phenylene benzobisoxazole) Fibers

**DOI:** 10.3390/polym18172118

**Published:** 2026-08-31

**Authors:** Xiangyu Zhao, Difei Wang, Xueting Wang, Xiaohui He

**Affiliations:** Electric Power Research Institute, State Grid Heilongjiang Electric Power Company Limited, Harbin 150030, China

**Keywords:** epoxy resin, PBO fiber, SiO_2_ coating, dynamic covalent bond, thermal conductivity, dielectric properties, chemical degradation, recyclable thermosets

## Abstract

Epoxy resins are widely used in electronic packaging and electrical insulation, but their permanent cross-linked networks and low intrinsic thermal conductivity restrict material recycling and heat dissipation. In this work, a recyclable epoxy composite was developed by combining a dual-dynamic epoxy network with silica-coated poly(p-phenylene benzobisoxazole) fibers. The composite architecture integrates thermally activated network rearrangement, amine-induced matrix degradation, electrically insulating fibrous heat transport, and recovery of the reinforcing phase within a single material system. The silica-containing coating modified the PBO-fiber surface morphology and fiber/matrix interphase, while increasing fiber content produced a progressive increase in thermal conductivity. At a fiber content of 12 wt%, the composite exhibited a thermal conductivity of 0.267 W/(m·K), together with low dielectric loss and a balance among thermal, dielectric, and mechanical properties. After hot-press reprocessing, the composite retained 92.1% of its original thermal conductivity. Immersion in n-hexylamine resulted in macroscopic disintegration of the epoxy matrix, enabling recovery of PBO@SiO_2_ fibers that retained their overall morphology and surface Si distribution. These results establish the composition–property relationships of a recyclable, thermally conductive, and electrically insulating fiber-reinforced epoxy composite. This work provides a feasible strategy for designing recyclable epoxy composites with balanced thermal conductivity, dielectric reliability, and fiber recoverability.

## 1. Introduction

Epoxy resin is a typical thermosetting polymer with excellent adhesion, dimensional stability, chemical resistance, mechanical strength, and electrical insulation properties. It has been widely used in electronic packaging, electrical insulation, coatings, adhesives, and fiber-reinforced composites [1,2]. However, a permanent three-dimensional cross-linked network is formed during curing, making epoxy resins difficult to melt, dissolve, or reprocess after service. As a result, end-of-life epoxy materials are generally disposed of by landfilling, incineration, mechanical grinding, or energy-intensive chemical treatment [3,4]. These approaches not only make it difficult to achieve high-value recovery of the resin matrix but also cause resource waste and environmental burdens. Therefore, developing new epoxy resin systems that combine service stability with recyclability has become an important research direction for sustainable thermosetting polymers [5,6].

In recent years, covalent adaptable networks and dynamic covalent chemistry have provided effective routes for designing recyclable epoxy resins [7,8]. By introducing reversible dynamic bonds into epoxy cross-linked networks, the materials can undergo bond exchange or bond cleavage/reformation under external stimuli such as heat, light, chemical reagents, or pH changes, thereby enabling stress relaxation, hot-press reprocessing, damage repair, or chemical degradation [9,10]. Among various dynamic bonds, disulfide bonds and imine bonds have attracted considerable attention in recyclable epoxy resins because of their relatively mild exchange conditions, high response efficiency, and generally catalyst-free exchange behavior [11]. Disulfide bonds can undergo reversible exchange reactions under thermal stimulation, which is beneficial for network topology rearrangement and hot-press reshaping [12]. Imine bonds can induce network depolymerization through imine exchange, hydrolysis, or aminolysis reactions, thereby providing a structural basis for chemical degradation and recycling [13,14]. In addition, bio-based aromatic aldehydes such as vanillin can react with diamines to form Schiff-base structures containing imine bonds and can further participate in epoxy curing reactions, providing a feasible strategy for constructing bio-based, reprocessable, and degradable epoxy resins [15].

Although dynamic covalent bonds can effectively improve the recyclability of epoxy resins, most recyclable epoxy systems still suffer from insufficient thermal conductivity. The intrinsic thermal conductivity of epoxy resin is generally only 0.17–0.20 W/(m·K), mainly because of the disordered arrangement of amorphous molecular chains, inefficient segmental vibration transfer, and severe phonon scattering [16,17]. With the development of electronic devices toward high integration and high power density, packaging and insulating materials require not only sustainable recyclability but also improved heat conduction capability to reduce local heat accumulation and enhance device reliability [18,19]. At present, a common method for improving the thermal conductivity of epoxy resins is to introduce thermally conductive fillers, such as boron nitride, alumina, graphene, carbon nanotubes, and silica [20]. These fillers can construct phonon transport pathways in the resin matrix, thereby improving the thermal conductivity of composites. However, for electrical insulation and electronic packaging applications, high filler loadings often increase system viscosity, reduce processability, and may cause local electric-field distortion because of filler aggregation, interfacial defects, and dielectric mismatch, leading to increased dielectric loss or reduced breakdown strength [21]. Therefore, balancing thermal conductivity enhancement, electrical insulation reliability, and reprocessability in recyclable epoxy resins remains challenging.

Poly(p-phenylene benzobisoxazole) (PBO) fiber is a high-performance organic fiber with high strength, high modulus, excellent heat resistance, a low dielectric constant, and relatively high axial thermal conductivity. These characteristics make PBO fibers promising reinforcements for high-performance structural composites, electrical-insulation components, and thermally managed electronic-packaging materials [22,23]. In the context of the present study, PBO fibers are considered particularly suitable for recyclable epoxy-based insulating components that require heat dissipation, dielectric reliability, and mechanical integrity simultaneously. Compared with particulate inorganic fillers, fibrous reinforcements are more likely to form continuous or semi-continuous thermal-transport pathways in resin matrices, which is beneficial for improving thermal conductivity at relatively low loadings. Meanwhile, PBO fibers possess good insulating characteristics, thereby avoiding the increase in dielectric loss that may be caused by conductive fillers [24,25]. However, PBO fibers have chemically inert surfaces and few polar functional groups, resulting in weak interfacial bonding with epoxy matrices. This may lead to interfacial voids, debonding, and stress concentration, thereby increasing interfacial thermal resistance and reducing the mechanical properties and breakdown reliability of the composites [26]. Therefore, improving the PBO-fiber/epoxy interface is important for exploiting the thermal and structural reinforcement potential of PBO fibers.

For the above application scenarios, SiO_2_-coated PBO (PBO@SiO_2_) fibers are intended to serve as surface-engineered, electrically insulating reinforcements in recyclable epoxy composites used for electronic packaging and electrical insulation. Their proposed function is to promote heat transport and mechanical load transfer while maintaining a low dielectric response and allowing recovery of the fibrous phase after degradation of the epoxy matrix. Silica surface coating has been explored as a strategy for modifying the interface between PBO fibers and epoxy resin [24]. Constructing a SiO_2_-containing coating on PBO fibers through a sol–gel reaction can increase the surface roughness and polarity of the fibers and may improve resin wettability and the local fiber/matrix contact [27]. Such an interfacial layer is expected to reduce gross interfacial defects and interfacial thermal resistance, thereby facilitating heat transfer across the fiber/matrix interface. In addition, SiO_2_ possesses good electrical-insulation characteristics and thermal stability. The coating may therefore help reduce local electric-field concentration associated with interfacial defects or dielectric mismatch, contributing to the insulation reliability of the composite [28]. Furthermore, if the PBO@SiO_2_ fibers retain their structural integrity during degradation of the recyclable epoxy matrix, separation and recovery of the reinforcing phase may be achieved, providing an additional benefit for the sustainable use of thermosetting composites [29,30].

Based on the above considerations, a recyclable PBO-fiber-reinforced epoxy composite was constructed using a single matrix formed by co-curing VOEP and DGEBA with 2-AFD. VOEP contains preformed imine linkages, whereas DGEBA supplies conventional glycidyl ether epoxy groups. The active amine hydrogens of 2-AFD react with the epoxy groups of both components, incorporating disulfide linkages into the cured network. The PBO fibers were coated with a silica-containing layer through a sol–gel process to increase their surface roughness and modify the fiber/epoxy interphase. The effects of PBO@SiO_2_ fiber content on the microstructure, dielectric properties, breakdown strength, thermal conductivity, thermal stability, and mechanical properties of the composites were systematically investigated. Furthermore, the hot-press reprocessability, n-hexylamine-induced chemical degradation behavior, and structural retention of PBO@SiO_2_ fibers after degradation were evaluated. This work aims to achieve a balance among thermal conductivity enhancement, insulation reliability, and sustainable recycling performance through the integrated design of a dual-dynamic network and surface-modified organic fibers, providing a reference for recyclable thermosetting composites used in electronic packaging and electrical insulation.

## 2. Materials and Methods

### 2.1. Materials

Bisphenol A diglycidyl ether (DGEBA, ≥85%), vanillin (VAN, ≥99%), 4,4′-oxydianiline (ODA, 98%), ethyl acetate (EAC, ≥99.8%), epichlorohydrin (ECH, 99%), tetrabutylammonium bromide (TBAB, 99%), sodium hydroxide aqueous solution (NaOH, 40 wt%), 2,2′-diaminodiphenyl disulfide (2-AFD, ≥98%), ammonia solution (NH_3_·H_2_O, 25 wt%), and n-hexylamine (C_6_H_15_N, 99%) were purchased from Beijing Inokai Technology Co., Ltd. (Beijing, China). Tetraethyl orthosilicate (TEOS, 99%) and cetyltrimethylammonium bromide (CTAB, 99%) were purchased from Shanghai Aladdin Biochemical Technology Co., Ltd. (Shanghai, China). Anhydrous ethanol and deionized water were used as solvents or washing agents. Poly(p-phenylene benzobisoxazole) fibers (PBO fibers) were supplied by Chengdu Xinchen New Materials Technology Co., Ltd. (Chengdu, China). The fibers used in this study had a length of approximately 5 mm and a diameter of approximately 12 μm, corresponding to an aspect ratio (L/D) of approximately 420. According to the available product specifications, the PBO fibers have a PBO content of approximately ≥99 wt%, a density of approximately 1.54 g cm^−3^, a tensile strength of approximately 5.8 GPa, and a tensile modulus in the range of approximately 160–280 GPa. The fibers also exhibit a decomposition temperature of approximately 650 °C and a limiting oxygen index of approximately 68%. The fibers were used without further cutting before the SiO_2_-coating treatment. All reagents were used as received without further purification unless otherwise stated.

### 2.2. Synthesis of the Vanillin-Based Schiff-Base Precursor (VO)

The vanillin-based Schiff-base precursor containing imine bonds was synthesized through a condensation reaction between VAN and ODA. A total of 20.024 g of ODA was first added into 100 mL of anhydrous ethanol in a flask equipped with a reflux condenser and magnetic stirring. The mixture was heated to 75 °C and stirred until ODA was completely dissolved. Separately, 30.43 g of VAN was dissolved in 50 mL of anhydrous ethanol at room temperature to obtain a homogeneous VAN solution.

The VAN solution was then added dropwise to the ODA solution within 25 min under continuous stirring. After the addition was completed, the reaction mixture was maintained at 75 °C for 2 h. A yellow precipitate gradually formed during the reaction. The product was collected by filtration, washed with ethanol to remove residual reactants, and dried in an oven at 60 °C to obtain the vanillin-based Schiff-base precursor, denoted as VO. The yield of VO was approximately 95%.

### 2.3. Synthesis of the Vanillin-Based Epoxy Monomer (VOEP)

**Figure 1:**
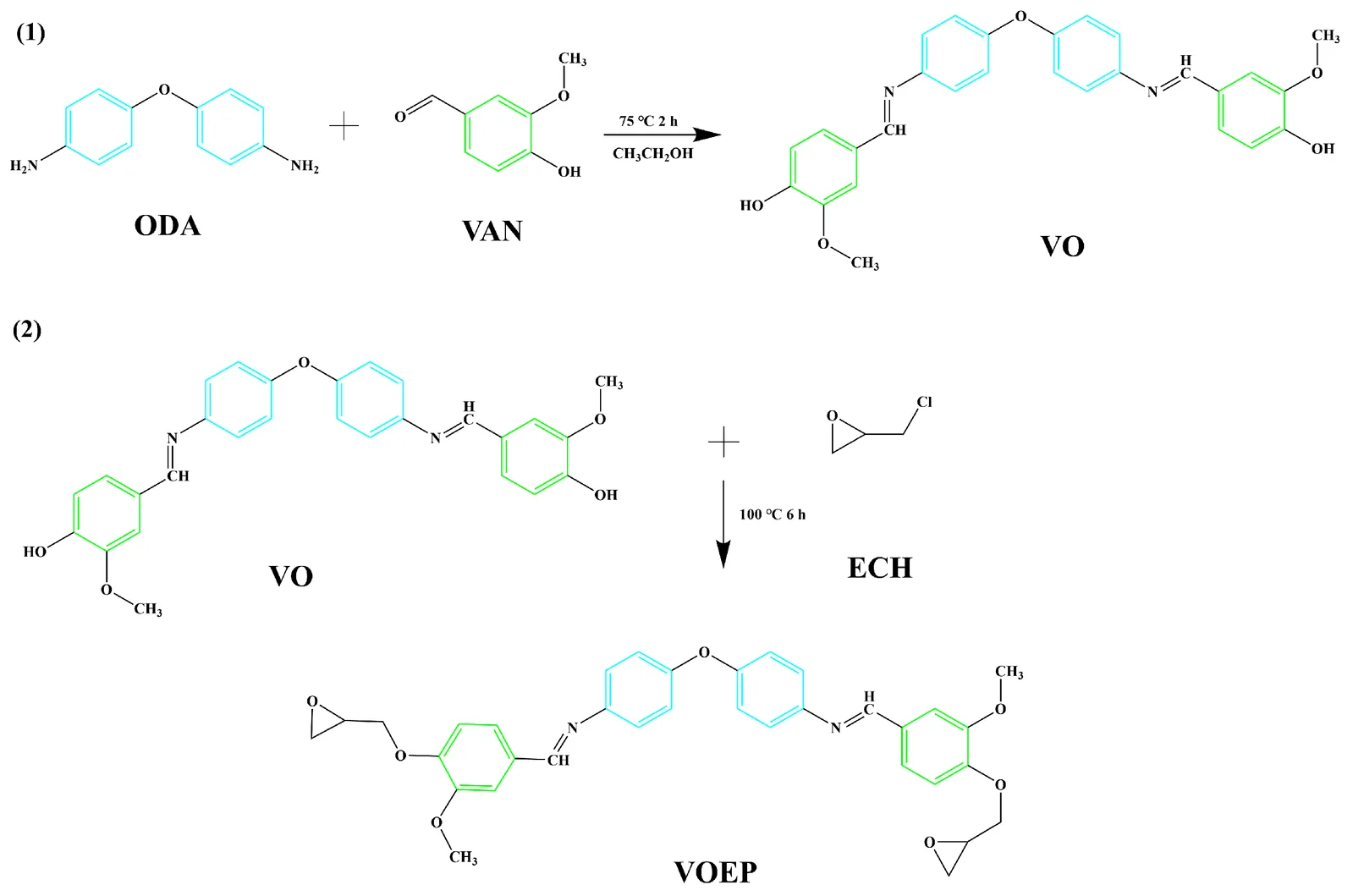
Sequential synthesis of VOEP: (**1**) Schiff-base condensation of vanillin with 4,4′-oxydianiline to form the isolated imine-containing precursor VO and (**2**) epoxidation of the phenolic hydroxyl groups of VO with epichlorohydrin to obtain VOEP.

As shown in Figure 1, VOEP was prepared by the epoxidation of VO with ECH. Briefly, 20 g of VO, 60 g of ECH, and 3 g of TBAB were placed into a flask equipped with mechanical stirring. The mixture was heated in an oil bath at 100 °C for 6 h. After the reaction, the temperature was decreased to 75 °C, and 30 mL of NaOH aqueous solution was slowly added dropwise. The reaction was continued for another 30 min.

After cooling to room temperature, the resulting light-yellow solid was collected by filtration and purified by recrystallization using ethyl acetate. The purified solid was washed three times with deionized water to remove residual salts and impurities, and then dried in an oven at 65 °C. The obtained product was denoted as VOEP. The yield of VOEP was approximately 78.6%.

### 2.4. Preparation of SiO_2_-Coated PBO Fibers

SiO_2_-coated PBO fibers were prepared through a sol–gel process. First, 20 mL of TEOS and 20 mL of anhydrous ethanol were mixed and stirred at room temperature for 20 min to form solution A. Separately, 0.2 g of CTAB was dispersed in 1 L of deionized water and stirred for 10 min. Then, 10 mL of 25 wt% aqueous ammonia solution was added, and the mixture was further stirred to obtain solution B.

10 g of PBO fibers were added into solution B and dispersed under stirring. Solution A was then slowly added dropwise into the PBO fiber suspension. After the addition was completed, the reaction mixture was heated to 60 °C and maintained under stirring for 12 h. The treated fibers were collected by filtration and washed three times with anhydrous ethanol and deionized water to remove unreacted precursors and physically adsorbed species. Finally, the fibers were dried in a vacuum oven at 60 °C for 24 h to obtain SiO_2_-coated PBO fibers, denoted as PBO@SiO_2_.

### 2.5. Preparation of PBO@SiO_2_/EP Composites

The VOEP/DGEBA/2-AFD formulation was designed as a single co-cured epoxy matrix. VOEP supplied preformed imine linkages associated with amine-induced network degradation, whereas DGEBA provided conventional glycidyl ether epoxy groups for the formation of the co-cured network. During curing, 2-AFD served as the curing agent and introduced disulfide linkages that enabled thermally activated network rearrangement. The SiO_2_-containing coating on the PBO fibers was used to increase fiber surface roughness and regulate fiber/epoxy interfacial contact. The DGEBA/2-AFD system was prepared only as a control to distinguish the contribution of VOEP and the resulting imine/disulfide dual-dynamic network.

The recyclable epoxy matrix was prepared using VOEP, DGEBA, and 2-AFD. The formulation was designed using a nominal 1:1 equivalent ratio between epoxy groups and active amine hydrogens. For the P-0% matrix, corresponding to a VOEP/DGEBA mass ratio of 3:2, 6.789 g of VOEP, 4.526 g of DGEBA, and 3.105 g of 2-AFD were used, with the total amount of the two epoxy components corresponding to 0.025 mol. For comparison, the Pure reference sample was prepared using 8.511 g of DGEBA and 3.105 g of 2-AFD under the same stoichiometric criterion. DGEBA was first heated to 100 °C under stirring to reduce its viscosity. VOEP and 2-AFD were then added, and the mixture was heated to 140 °C and stirred for 30 min until a homogeneous resin system was obtained.

For the preparation of PBO@SiO_2_/EP composites, predetermined amounts of PBO@SiO_2_ fibers were first dispersed in anhydrous ethanol under mechanical stirring at 30 °C. Ethanol served as a temporary low-viscosity dispersion medium to wet the SiO_2_-coated fiber surfaces and separate fiber bundles, thereby facilitating the subsequent incorporation of the fibers into the viscous resin mixture and reducing local fiber aggregation. The ethanol was then removed by filtration, and the fibers were dried at 60 °C to remove residual solvent. The dried PBO@SiO_2_ fibers were gradually introduced into the homogeneous VOEP/DGEBA/2-AFD resin mixture. The mixture was further stirred to promote uniform fiber dispersion and then degassed in a vacuum oven at 120 °C for 1 h.

The degassed mixture was poured into a preheated mold and cured according to a stepwise curing procedure of 140 °C for 2 h followed by 160 °C for 3 h. After curing, the samples were naturally cooled to room temperature and demolded. According to the mass fraction of PBO@SiO_2_ fibers in the composites, the samples were denoted as P-0%, P-4%, P-8%, P-12%, and P-16%.

For comparison, a reference epoxy sample without VOEP and PBO@SiO_2_ fibers was also prepared by curing DGEBA with 2-AFD under the same stoichiometric ratio and curing procedure. This reference sample was denoted as Pure.

### 2.6. Physical Hot-Pressing Reprocessing

The reprocessability of the cured epoxy resins and composites was evaluated by physical hot pressing. The fractured specimens obtained after tensile testing were mechanically pulverized into small particles. The particles were then placed into a steel mold and hot-pressed using a flat vulcanizing press at 180 °C and 15 MPa for 4 h. After hot pressing, the mold was cooled to room temperature, and the reprocessed samples were demolded.

The reprocessed samples were denoted as Pure-Re, P-0%-Re, P-4%-Re, P-8%-Re, P-12%-Re, and P-16%-Re according to their original compositions.

### 2.7. Chemical Degradation and Fiber Recovery

Chemical degradation tests were conducted to evaluate the degradability of the recyclable epoxy matrix and the recoverability of PBO@SiO_2_ fibers. The cured samples were immersed in *n*-hexylamine solution at 90 °C. The degradation process was recorded at different time intervals. After degradation, the residual fibers were collected by filtration, washed with ethanol and deionized water, and dried at 60 °C.

The recovered fibers were further characterized by SEM and EDS to evaluate whether the PBO@SiO_2_ fiber morphology and SiO_2_ coating were retained after chemical degradation.

### 2.8. Characterizations

Fourier transform infrared spectroscopy (FTIR) was used to analyze the chemical structures of VAN, ODA, VO, VOEP, and the cured epoxy networks. The spectra were recorded on a Bruker FTIR spectrometer (Bruker Optics GmbH & Co. KG, Ettlingen, Germany) in the range of 4000–400 cm^−1^ using the KBr pellet method.

Proton nuclear magnetic resonance spectroscopy (^1^H NMR) was used to confirm the chemical structures of VO and VOEP. The spectra were collected on a Bruker AVANCE III 400 MHz spectrometer (Bruker BioSpin GmbH & Co. KG, Ettlingen, Germany) at 298 K using DMSO-d_6_ as the solvent. The spectral width was 12 ppm, the relaxation delay was 1 s, and the number of scans was 32.

Scanning electron microscopy (SEM) was performed using a Hitachi SU-9600N scanning electron microscope (Hitachi High-Tech Corporation, Tokyo, Japan) to observe the morphology of PBO fibers, PBO@SiO_2_ fibers, fractured surfaces of the composites, and recovered fibers after chemical degradation. Before observation, the samples were fractured to expose fresh cross-sections and sputter-coated with gold for 120 s. Energy-dispersive X-ray spectroscopy (EDS) was used to analyze the elemental distribution on the fiber surfaces.

Dielectric properties were measured using a Novocontrol Concept 40 broadband dielectric spectrometer (Novocontrol Technologies GmbH & Co. KG, Montabaur, Germany) at 25 °C over a frequency range of 10^1^–10^6^ Hz. Before testing, circular aluminum electrodes with a diameter of 6 mm were deposited on both sides of each sample by vacuum evaporation to ensure good electrical contact.

AC breakdown strength was measured using a column-plate electrode system connected to a high-voltage AC power supply and a YDK transformer control unit. Rectangular film samples with dimensions of approximately 5 cm × 10 cm × 200 μm were used for the test. The voltage was increased at a constant rate until electrical breakdown occurred, and the breakdown voltage was recorded. At least 10 valid breakdown data points were collected for each sample. The characteristic breakdown strength was analyzed using a two-parameter Weibull distribution.

Thermal conductivity was measured using a DRL-III heat-flow thermal conductivity tester (Xiangtan Xiangyi Instrument Co., Ltd., Xiangtan, China) according to ASTM D5470 [31]. The upper and lower copper electrodes were maintained at 75 °C and 25 °C, respectively. The applied pressure was 100 N, and the stabilization time was 120 s. Circular samples with a diameter of 2 cm and a thickness of approximately 200 μm were used.

Thermogravimetric analysis (TGA) was performed to evaluate the thermal stability of the samples. Approximately 5 mg of each cured sample was placed in the sample crucible and heated from 35 °C to 900 °C under an argon atmosphere at a heating rate of 15 °C/min. The TGA and derivative thermogravimetric curves were recorded. The residual mass at 900 °C was used as the char yield.

Tensile properties were tested using an Instron 5567 universal testing machine (Instron, Norwood, MA, USA) ac-cording to ASTM D638 [32]. Dumbbell-shaped specimens were stretched at a crosshead speed of 5 mm/min at room temperature. At least five specimens were tested for each formulation. The results are presented as mean ± standard deviation, and the error bars represent the standard deviation.

For the supplementary characterizations, X-ray photoelectron spectroscopy (XPS) was performed using a Kratos AXIS Ultra DLD instrument (Kratos Analytical Ltd., Manchester, UK) to determine the surface elemental composition and Si 2p chemical state of pristine PBO and PBO@SiO_2_ fibers; the binding-energy scale was referenced to the C 1s peak at 284.8 eV. Differential scanning calorimetry (DSC) was performed using a NETZSCH DSC 214 instrument (NETZSCH-Gerätebau GmbH, Selb, Germany) under nitrogen to evaluate the glass-transition and residual-curing behavior of P-0%, P-12%, and P-12%-Re. Dynamic mechanical analysis (DMA) was conducted using a NETZSCH DMA 303 Eplexor instrument to record the storage modulus and Tan*δ* as functions of temperature. Infrared thermography was performed using a Teledyne FLIR T450 camera (Teledyne FLIR, LLC, Wilsonville, OR, USA); P-0% and P-12% specimens were subjected to identical preheating, and their surface-temperature evolution during natural cooling was recorded. The finite-element comparisons used identical geometry and thermal boundary conditions to calculate the relative temperature and heat-flux fields of the comparative material models.

## 3. Results and Discussion

### 3.1. Structural Characterization of VOEP

**Figure 2:**
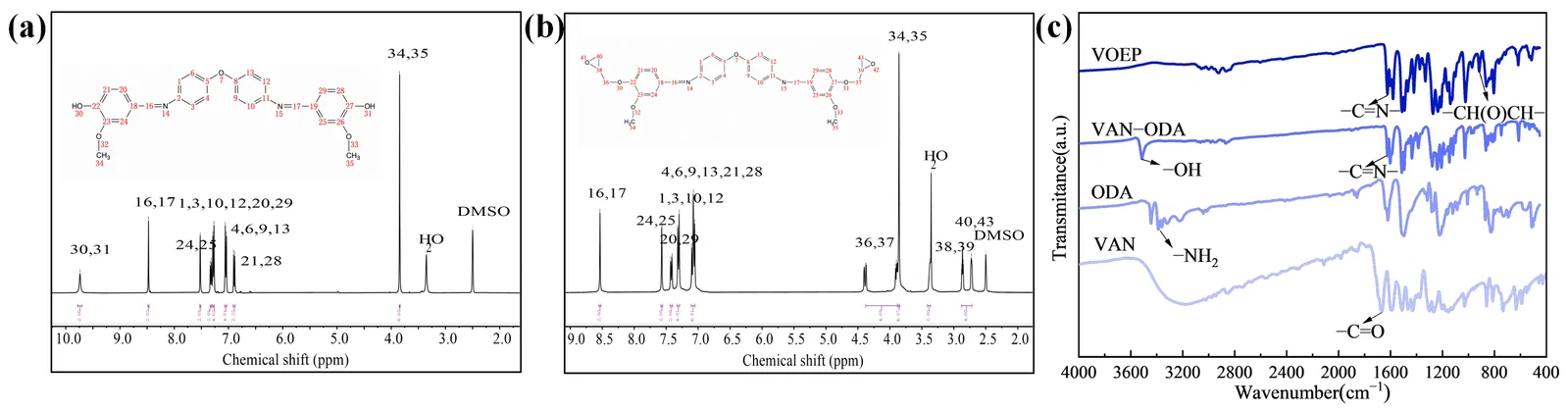
^1^H NMR and FTIR characterization of VO and VOEP: (**a**) ^1^H NMR spectrum of VO; (**b**) ^1^H NMR spectrum of VOEP; (**c**) FTIR spectra of VAN, ODA, VO, and VOEP.

Figure 2 shows the ^1^H NMR and FTIR characterization results of VO and VOEP. Figure 2a presents the ^1^H NMR spectrum of VO. A characteristic proton signal of the imine bond (-CH=N-) is observed at *δ* = 8.48 ppm, while the phenolic hydroxyl proton signal appears at *δ* = 9.74 ppm. These results indicate that the aldehyde group in vanillin reacted with the amino groups in ODA through a Schiff-base condensation reaction, successfully forming the imine-containing VO structure [33]. Meanwhile, the aromatic proton signals are mainly distributed in the range of *δ* = 6.89–7.52 ppm, and the methoxy proton signal appears at *δ* = 3.84 ppm, which is consistent with the expected molecular structure.

Figure 2b shows the ^1^H NMR spectrum of VOEP. Compared with VO, the phenolic hydroxyl proton signal near *δ* = 9.74 ppm disappears in VOEP, indicating that the phenolic hydroxyl groups in VO participated in the epoxidation reaction [34]. Meanwhile, proton signals associated with the glycidyl ether structure appear near *δ* = 4.38, 3.88, and 2.88 ppm, confirming that epoxy groups were successfully introduced into the VO molecular structure. The imine proton signal is still observed at *δ* = 8.54 ppm in VOEP, indicating that the epoxidation reaction did not destroy the dynamic imine structure. Therefore, VOEP contains both epoxy groups capable of participating in curing reactions and dynamic imine bonds with reversible characteristics.

Figure 2c shows the FTIR spectra of VAN, ODA, VO, and VOEP. The simultaneous disappearance of the aldehyde absorption of VAN near 1672 cm^−1^ and the primary amine absorption bands of ODA at 3212–3462 cm^−1^ indicates the consumption of both functional groups. Concurrently, a new C=N absorption band appears near 1622 cm^−1^, corresponding to the formation of imine linkages through Schiff-base condensation between VAN and ODA [35,36,37]. The imine proton signal observed at δ = 8.48 ppm in the ^1^H NMR spectrum is consistent with the assigned VO structure. For VOEP, the absorption peak assigned to phenolic hydroxyl groups in the range of 3480–3530 cm^−1^ nearly disappears, while a characteristic epoxy absorption peak appears near 914 cm^−1^, indicating that the phenolic hydroxyl groups in VO were successfully epoxidized by epichlorohydrin [38]. The ^1^H NMR and FTIR results collectively confirm the successful synthesis of VOEP.

### 3.2. Microstructure and Interfacial Structure of PBO@SiO_2_/EP Composites

**Figure 3:**
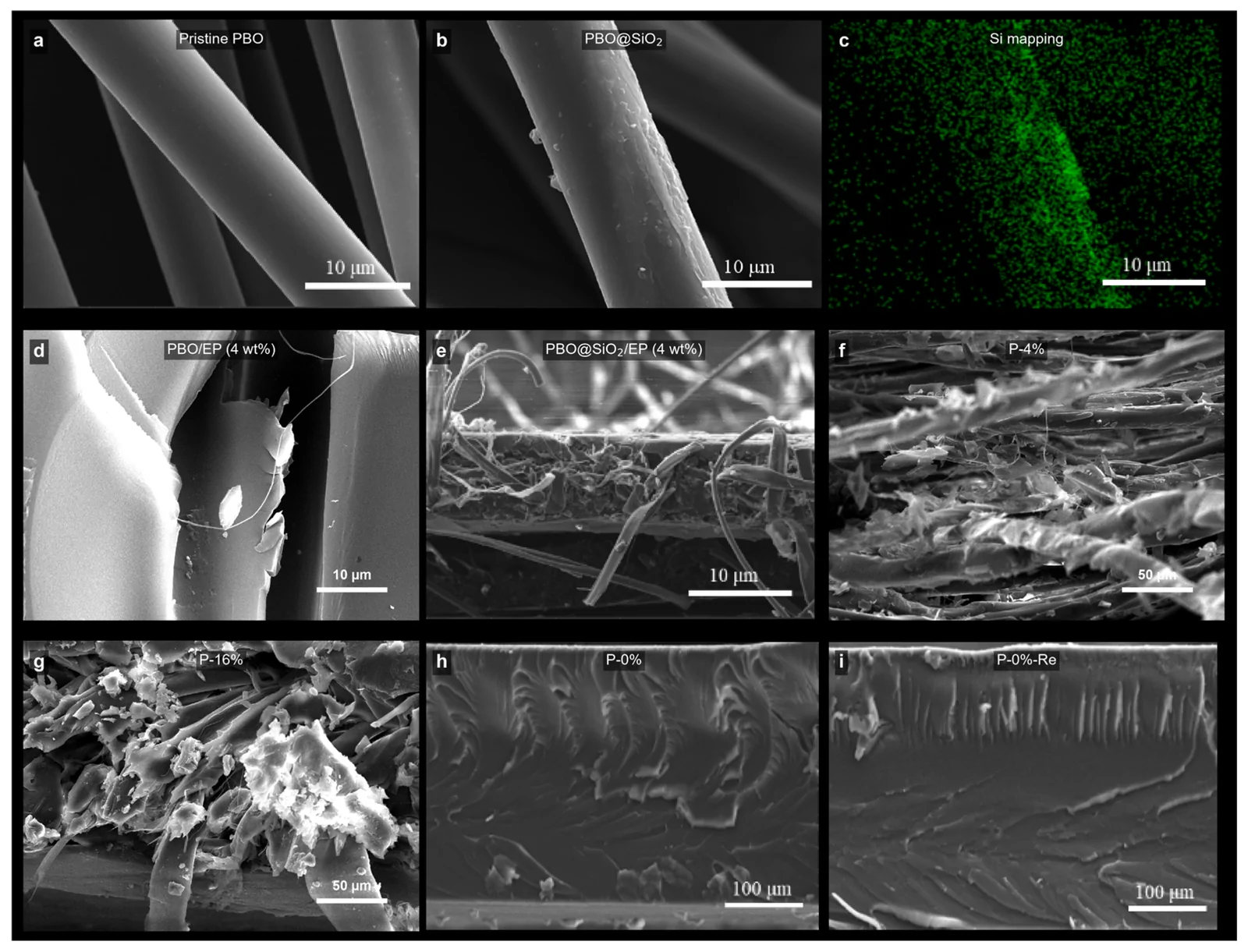
SEM images and EDS elemental mapping of the fibers and composites: (**a**) pristine PBO fibers; (**b**) PBO@SiO_2_ fibers; (**c**) Si elemental mapping of PBO@SiO_2_ fibers; (**d**) fracture surface of the composite reinforced with 4 wt% uncoated PBO fibers; (**e**) local fiber/matrix morphology of P-4%; (**f**,**g**) fracture surfaces of P-4% and P-16%, respectively; and (**h**,**i**) fracture surfaces of P-0% before and after hot-press reprocessing, respectively.

Figure 3 presents the surface morphology of the fibers and the fracture morphology of representative composites. Pristine PBO fibers exhibit relatively smooth surfaces (Figure 3a). After the TEOS-based sol–gel treatment, PBO@SiO_2_ fibers show a rougher surface covered with particulate features (Figure 3b), and Si is distributed along the fiber profile in the corresponding EDS map (Figure 3c). XPS provides complementary surface-chemical evidence (Figure S2 and Table S1): after treatment, the O and Si contents increase from 17.02 to 25.21 at.% and from 3.32 to 5.80 at.%, respectively; the O/C and Si/C ratios increase from 0.228 and 0.045 to 0.376 and 0.086, respectively; and the Si 2p binding energy shifts from 102.15 to 103.15 eV. Together, the SEM/EDS and XPS results support the formation of a silica-rich surface layer on the PBO fibers.

Figure 3d shows the fracture surface of the composite reinforced with 4 wt% uncoated PBO fibers, whereas Figure 3e shows the local fiber/matrix morphology of P-4%. In Figure 3e, the coated fibers are embedded in the epoxy matrix without obvious large-area interfacial gaps within the selected field of view. The coated/uncoated comparisons in Figure S7 further show generally higher thermal conductivity and tensile/flexural strength for PBO@SiO_2_/EP than for PBO/EP at matched fiber contents. These comparative property trends, together with the surface-chemical and morphological evidence, are consistent with a beneficial coating-related interphase effect [39].

Figure 3f,g compare the fracture surfaces of P-4% and P-16%, respectively. The P-4% sample exhibits a comparatively uniform fracture morphology, whereas the higher-loading P-16% sample shows increased structural heterogeneity and locally concentrated fiber-rich regions. These observations are consistent with a balance between effective short-fiber reinforcement at low loading and the increasing influence of dispersion heterogeneity, porosity, and fiber/matrix interfacial density at high loading.

Figure 3h,i show the fracture surfaces of P-0% before and after hot-press reprocessing, respectively. The reprocessed sample retains a compact local fracture morphology, consistent with particle-interface reconstruction promoted by thermally activated exchange in the imine- and disulfide-containing network. Supplementary DSC measurements show glass-transition temperatures of 128.06, 121.46, and 120.17 °C for P-0%, P-12%, and P-12%-Re, respectively, with no distinct residual-curing exotherm apparent within the displayed temperature interval (Figure S3). DMA further shows the temperature-dependent decrease in storage modulus and the associated tan δ relaxation, while the P-12%-Re response remains broadly comparable to that of P-12% (Figure S4).

The morphology and Si elemental distribution of the PBO@SiO_2_ fibers recovered from P-12% after n-hexylamine-induced degradation at 90 °C for 4 h are presented in Figures S1c and S1d, respectively. The recovered fibers retain a continuous fibrous morphology and rough particulate surface features, while Si remains distributed along the fiber profile. These observations indicate that the silica-containing surface layer is largely retained during matrix degradation and support the effective recovery of PBO@SiO_2_ fibers.

These results show that the prepared PBO@SiO_2_/EP composites can be hot-press reprocessed through the dynamic network and can also undergo controlled matrix degradation and effective fiber recovery in n-hexylamine. Additional fracture-surface images of Pure and Pure-Re are provided in Figure S1a and Figure S1b, respectively.

### 3.3. Dielectric Properties and Breakdown Strength of PBO@SiO_2_/EP Composites

**Figure 4:**
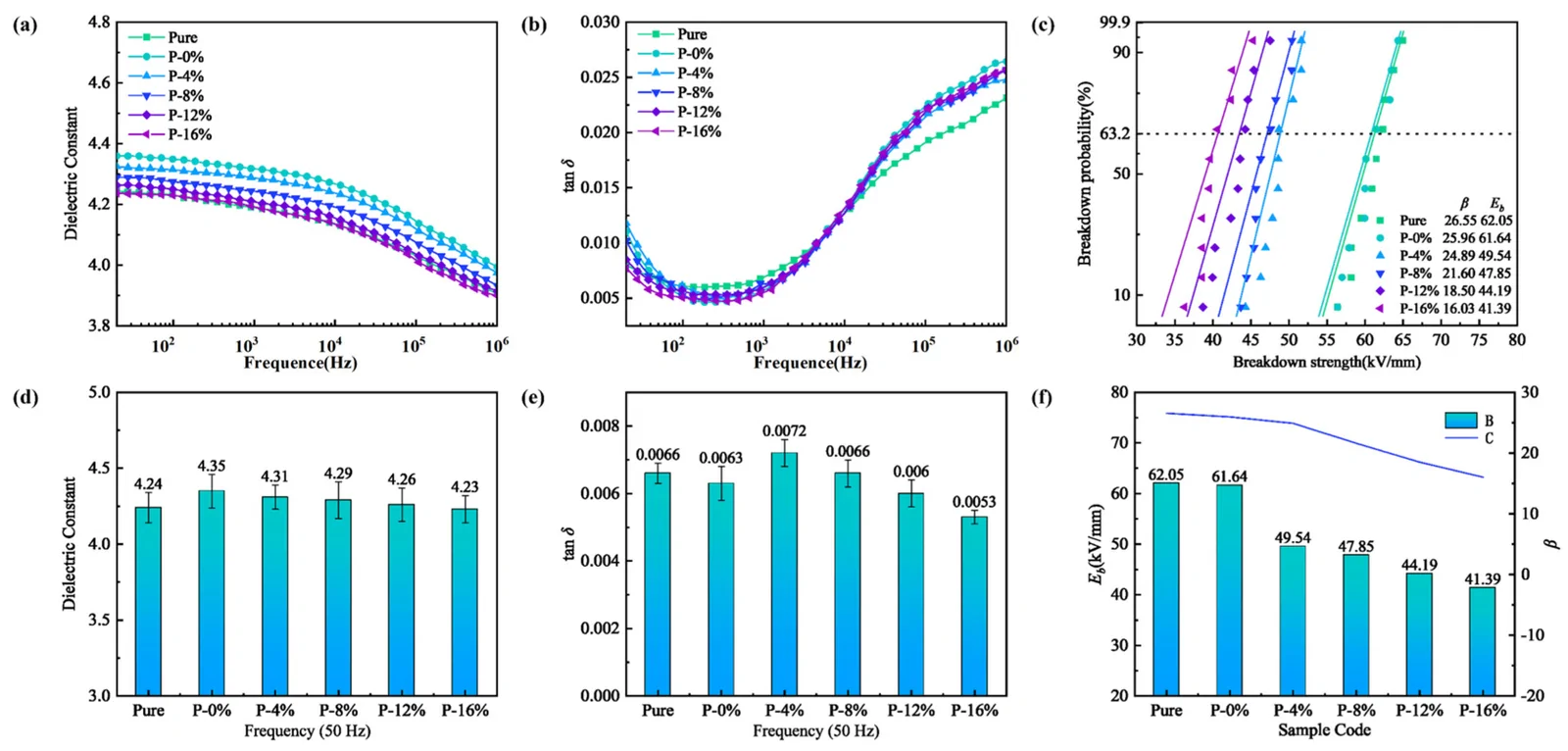
Dielectric and breakdown properties of epoxy resin composites before reprocessing: (**a**) frequency-dependent dielectric constant; (**b**) frequency-dependent dielectric loss; (**c**) Weibull distribution of breakdown strength; the horizontal dashed line indicates a cumulative breakdown probability of 63.2%, at which the characteristic breakdown strength is defined; (**d**) dielectric constant at 50 Hz; (**e**) dielectric loss at 50 Hz; (**f**) characteristic breakdown strength and Weibull shape parameter.

Figure 4 shows the dielectric and breakdown properties of the epoxy resins and PBO@SiO_2_/EP composites before reprocessing. As shown in Figure 4a,b, the dielectric constants of all samples gradually decrease with increasing frequency. This is because molecular segments and polar groups have sufficient time to respond to the applied electric field at low frequencies, resulting in a relatively large contribution from dipole orientation polarization. As the frequency increases, the dipole orientation gradually lags behind the change in the electric field, weakening the polarization response and reducing the dielectric constant.

Figure 4d shows the effect of fiber content on the dielectric constant. At 50 Hz, the dielectric constants of Pure, P-0%, P-4%, P-8%, P-12%, and P-16% are 4.24, 4.35, 4.31, 4.29, 4.26, and 4.23, respectively. The dielectric constant of P-0% is slightly higher than that of Pure, mainly because the introduction of VOEP brings polar structures such as imine bonds, aromatic groups, and ether bonds. With increasing PBO@SiO_2_ fiber content, the dielectric constant of the composites gradually decreases. This is because PBO fibers have a relatively low dielectric constant, which lowers the overall polarizability of the composite system [40]. Meanwhile, the SiO_2_ coating regulates the fiber/matrix interphase, reducing unstable interfacial polarization caused by defects and allowing the composites to maintain a stable dielectric response at higher fiber contents.

Figure 4e shows the effect of fiber content on dielectric loss. At 50 Hz, the dielectric losses of Pure, P-0%, P-4%, P-8%, P-12%, and P-16% are 0.0066, 0.0063, 0.0072, 0.0066, 0.0060, and 0.0053, respectively, all of which remain at low levels. These results indicate that the introduction of PBO@SiO_2_ fibers does not form obvious conductive pathways in the resin and does not significantly increase conductive loss [41]. For electrical insulation and electronic packaging materials, low dielectric loss helps reduce dielectric heating and energy dissipation during service. Therefore, PBO@SiO_2_/EP composites still show promising insulation potential.

Figure 4c,f show the Weibull distributions of the breakdown strength before reprocessing. The breakdown strength was analyzed using a two-parameter Weibull distribution. The characteristic breakdown strengths of Pure and P-0% are 62.05 and 61.64 kV/mm, respectively, indicating that the introduction of VOEP provides dynamic recyclable structures without significantly weakening the breakdown strength of the resin matrix. With increasing PBO@SiO_2_ fiber content, the breakdown strengths of P-4%, P-8%, P-12%, and P-16% decrease to 49.54, 47.85, 44.19, and 41.39 kV/mm, respectively. The decrease in breakdown strength is mainly attributed to the increased number of interfaces after fiber incorporation. Despite the surface modification, dielectric mismatch still exists, dielectric mismatch still exists between the fibers and the resin, which can induce local electric-field distortion under an applied electric field. Meanwhile, high fiber contents may increase the probability of micropores, local aggregation, and interfacial defects, which can act as weak points for electrical breakdown [42,43,44].

**Figure 5:**
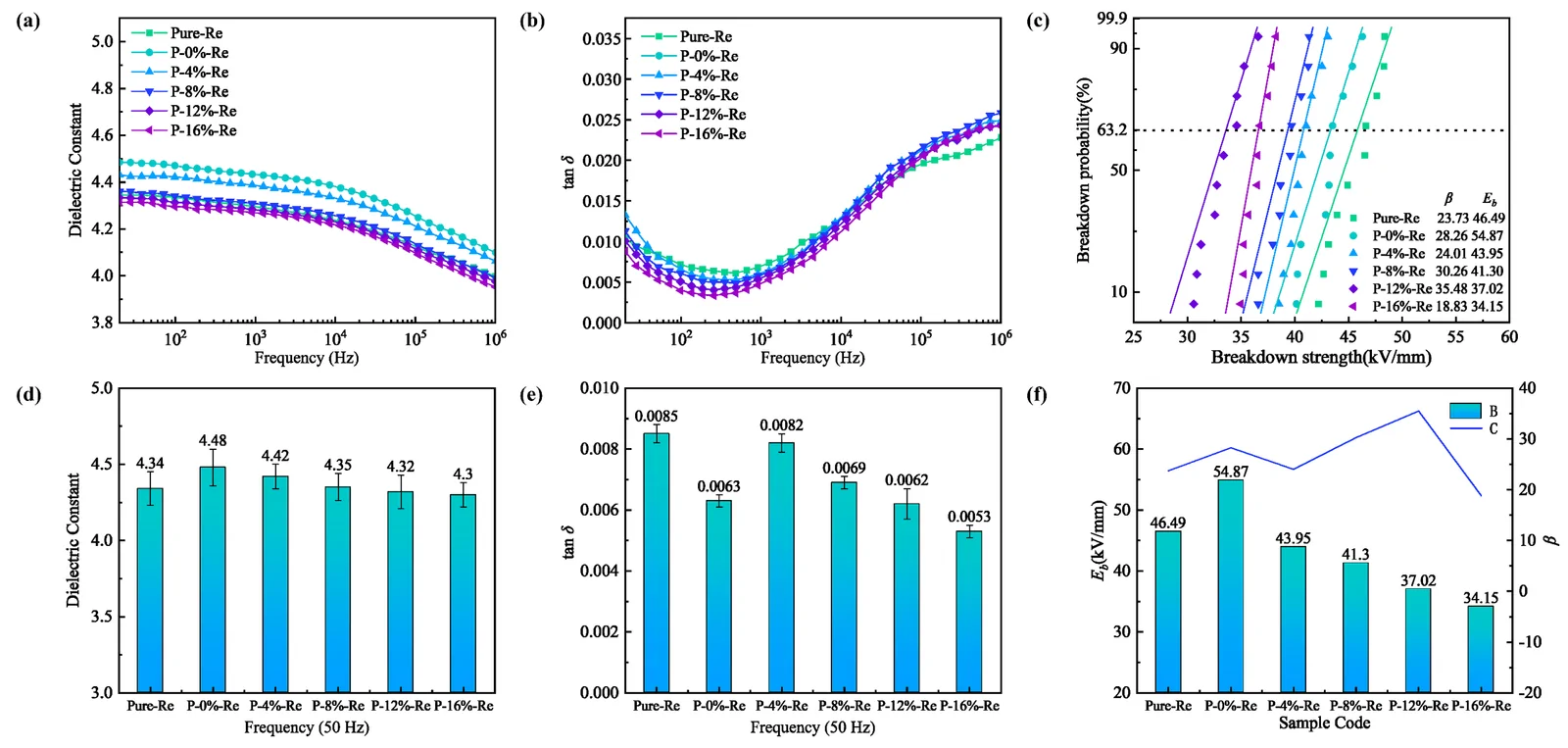
Dielectric and breakdown properties of epoxy resin composites after hot-press reprocessing: (**a**) frequency-dependent dielectric constant; (**b**) frequency-dependent dielectric loss; (**c**) Weibull distribution of breakdown strength; the horizontal dashed line indicates a cumulative breakdown probability of 63.2%, at which the characteristic breakdown strength is defined; (**d**) dielectric constant at 50 Hz; (**e**) dielectric loss at 50 Hz; (**f**) characteristic breakdown strength and Weibull shape parameter.

Figure 5 shows the dielectric and breakdown properties of the hot-press-reprocessed samples. As shown in Figure 5a,d, the dielectric constants of P-0%-Re, P-4%-Re, P-8%-Re, P-12%-Re, and P-16%-Re at 50 Hz are 4.48, 4.42, 4.35, 4.32, and 4.30, respectively, which are slightly higher than those of the corresponding samples before reprocessing. This phenomenon may be related to new interfaces formed during grinding and hot-press remolding. Particle interfaces and a small number of microscopic defects can enhance interfacial polarization, leading to a slight increase in dielectric constant. Overall, the dielectric constants of the reprocessed samples still decrease with increasing fiber content, indicating that the regulation effect of PBO@SiO_2_ fibers on the dielectric response is retained after reprocessing.

Figure 5b,e show the frequency-dependent dielectric loss and the dielectric loss at 50 Hz as a function of fiber content for the reprocessed composites. At 50 Hz, the dielectric losses of the reprocessed samples are 0.0063, 0.0082, 0.0069, 0.0062, and 0.0053, respectively, and remain at low levels. In particular, the dielectric losses of P-12%-Re and P-16%-Re are close to those before reprocessing, indicating that hot-press reprocessing does not significantly damage the low-loss characteristics of the materials. Figure 5c,f show the Weibull distributions of the reprocessed composites. The breakdown strengths of P-0%-Re, P-4%-Re, P-8%-Re, P-12%-Re, and P-16%-Re are 54.87, 43.95, 41.30, 37.02, and 34.15 kV/mm, respectively. Compared with the original samples, the breakdown strengths decrease, mainly because grinding and remolding inevitably introduce new particle interfaces and microscopic defects. Nevertheless, the breakdown strength retention of P-12%-Re still reaches 83.8%, indicating that the dual-dynamic network promotes network rearrangement and interfacial reconstruction during hot pressing, enabling the material to retain good insulation reliability after reprocessing.

The decrease in breakdown strength after reprocessing can be considered at two structural levels. Mechanical grinding converts the continuous cured network into discrete particles, which are subsequently rejoined through dynamic-bond exchange during hot pressing, thereby introducing particle–particle boundaries into the reconstructed material. Local discontinuities remaining at these boundaries can cause nonuniform electric-field distributions and provide preferential sites for breakdown initiation. The breakdown-strength retention of P-0%-Re, which contains no PBO@SiO_2_ fibers, is approximately 89.0%, showing that reprocessing of the epoxy matrix itself affects the local breakdown-limiting structure. In the fiber-containing composites, the reprocessed particle boundaries coexist with PBO@SiO_2_ fiber/matrix interfaces, and the difference in dielectric properties between the fibers and epoxy matrix makes the local electric-field distribution sensitive to the number and spatial arrangement of these interfaces. The breakdown-strength retention decreases progressively from 89.0% for P-0%-Re to 88.7%, 86.3%, 83.8%, and 82.5% for P-4%-Re, P-8%-Re, P-12%-Re, and P-16%-Re, respectively. This trend is consistent with an increasing contribution of fiber/matrix interfaces to local electrical heterogeneity as the fiber content increases. Therefore, the relatively stable dielectric constant and dielectric loss after reprocessing reflect the retention of the average dielectric response, whereas the reduction in breakdown strength reflects changes in the local regions controlling electrical failure.

### 3.4. Thermal Conductivity of PBO@SiO_2_/EP Composites

**Figure 6:**
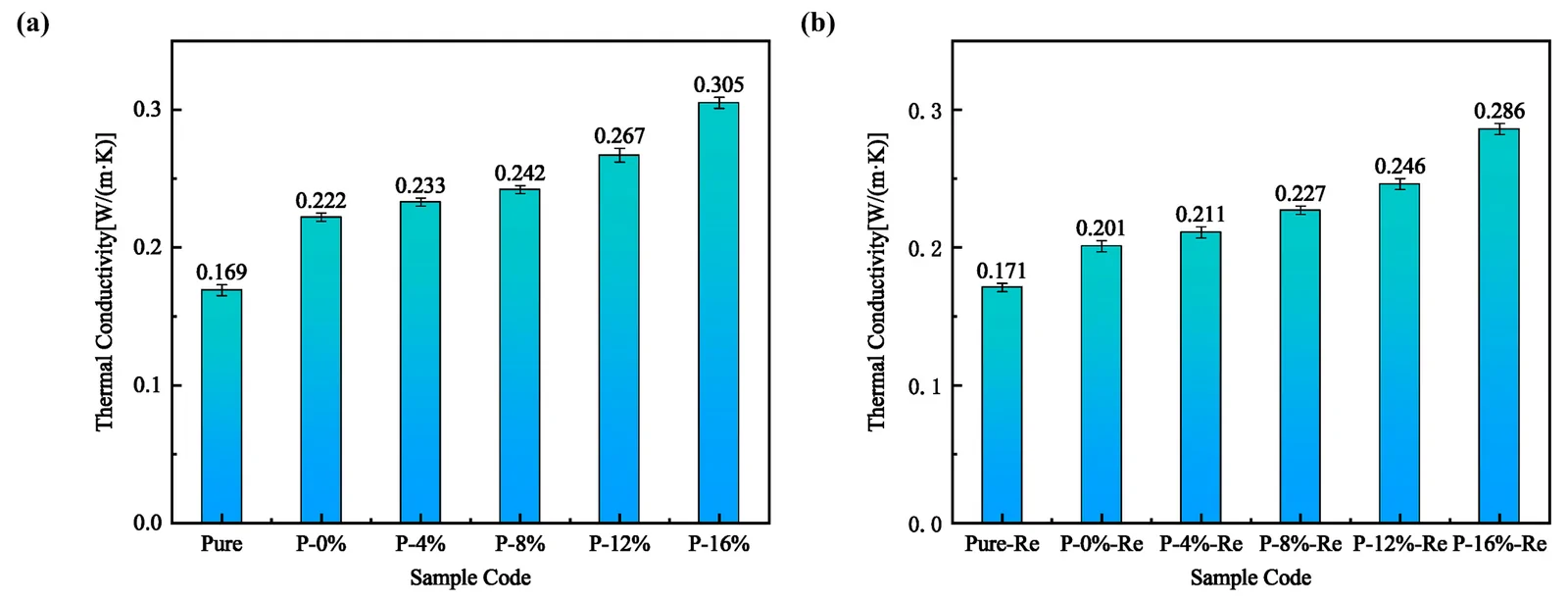
Thermal conductivity of epoxy resin composites with different PBO@SiO_2_ fiber contents: (**a**) samples before reprocessing; (**b**) samples after hot-press reprocessing.

Figure 6 shows the thermal conductivity of the composites before and after reprocessing. The thermal conductivity increases from 0.169 W/(m·K) for Pure to 0.222 W/(m·K) for P-0%. After incorporation of PBO@SiO_2_ fibers, the values of P-4%, P-8%, P-12%, and P-16% increase progressively to 0.233, 0.242, 0.267 and 0.305 W/(m·K), respectively. The larger increments between 8–12 wt% and 12–16 wt% are consistent with shorter interfiber separation and an increasing contribution of fiber proximity or contact. PBO fibers can serve as comparatively conductive axial heat-transport elements, while the silica-rich surface layer may modify the fiber/matrix interphase [45,46,47]. Importantly, the uncoated PBO/EP controls in Figure S7 exhibit lower thermal conductivity than the corresponding PBO@SiO_2_/EP composites at matched fiber contents, providing comparative support for a coating-related contribution.

Figure 6b further shows the thermal conductivity of the reprocessed composites. After reprocessing, the thermal conductivities of Pure-Re, P-0%-Re, P-4%-Re, P-8%-Re, P-12%-Re, and P-16%-Re are 0.171, 0.201, 0.211, 0.227, 0.246, and 0.286 W/(m·K), respectively. Compared with the original samples, the thermal conductivity of each composite slightly decreases, but it still increases with increasing fiber content. Taking P-12% as an example, the thermal conductivity of P-12%-Re is 0.246 W/(m·K), with a retention rate of 92.1%. The reprocessed samples still maintain relatively high thermal conductivity, indicating that the thermal conductive network constructed by PBO@SiO_2_ fibers is partially retained after hot-press reprocessing.

### 3.5. Thermal Stability of PBO@SiO_2_/EP Composites

To evaluate the heat resistance of the epoxy resins, the temperature index of heat resistance (*T*_HRI_) was calculated using Equation (1) [48]:

(1) $$T_{\mathrm{HRI}}=0.49\times (T_{5\%}+0.6\times (T_{30\%}-T_{5\%}))$$

**Figure 7:**
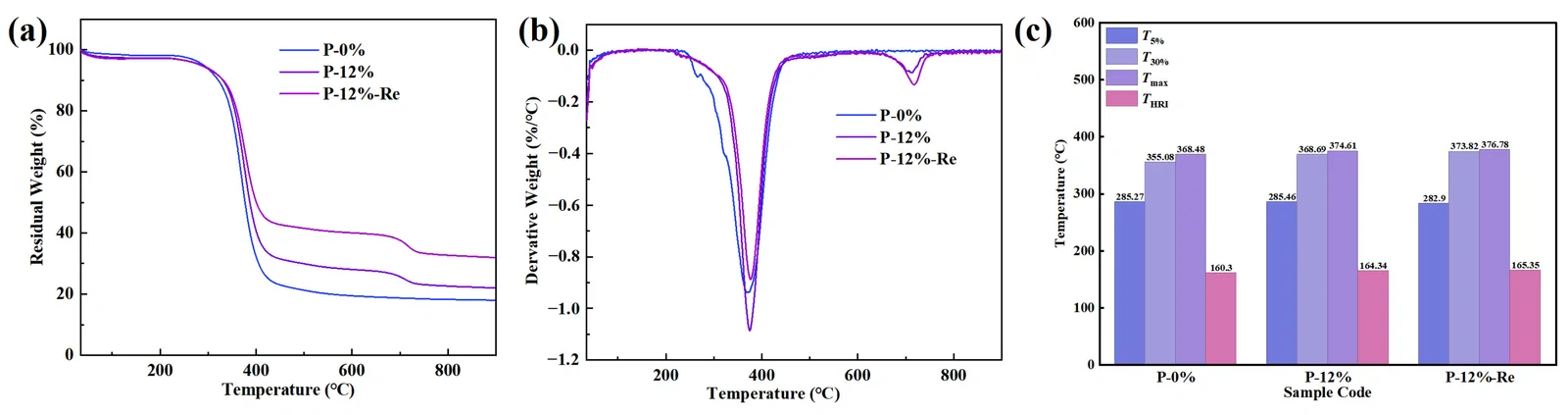
Thermal stability of P-0%, P-12%, and P-12%-Re: (**a**) TGA curves; (**b**) DTG curves; (**c**) thermal stability parameters, including *T*_5%_, *T*_30%_, *T*_max_, and *T*_HRI_.

Figure 7 shows the TGA and DTG curves and thermal stability parameters of P-0%, P-12%, and P-12%-Re. As shown in Figure 7a, the *T*_5%_ values of P-0%, P-12%, and P-12%-Re are 285.27, 285.46, and 282.90 °C, respectively, indicating that the introduction of PBO@SiO_2_ fibers has a limited effect on the initial decomposition temperature. The *T*_30%_ values of P-0%, P-12%, and P-12%-Re are 355.08, 368.69, and 373.82 °C, respectively. According to the DTG curves in Figure 7b, the *T*_max_ values are 368.48, 374.61, and 376.78 °C, respectively. Compared with P-0%, the *T*_30%_ and *T*_max_ of P-12% increase by 13.61 and 6.13 °C, respectively, indicating that PBO@SiO_2_ fibers significantly improve the thermal decomposition stability of the composites in the middle and late stages.

The *T*_HRI_ values calculated from the TGA data are 160.30, 164.34, and 165.35 °C for P-0%, P-12%, and P-12%-Re, respectively. Compared with P-0%, the *T*_HRI_ of P-12% increases by 4.04 °C, indicating that the incorporation of PBO@SiO_2_ fibers improves the heat resistance of the recyclable epoxy resin. This result can be attributed to two factors. First, PBO fibers have excellent thermal stability and can act as a thermally stable skeleton in the composite [49]. Second, the SiO_2_ coating on the fiber surface can serve as an inorganic thermal barrier, delaying heat transfer into the resin matrix and hindering the diffusion of some thermal decomposition products, thereby postponing the decomposition process [50,51].

In addition, the *T*_HRI_ of P-12%-Re is 165.35 °C, slightly higher than that of P-12%, indicating that the reprocessing procedure has little effect on the overall thermal stability of the composite. The thermal stability of the reprocessed sample is comparable to or slightly better than that of the original sample, demonstrating good recyclability and thermal stability retention.

### 3.6. Mechanical Properties of PBO@SiO_2_/EP Composites

**Figure 8:**
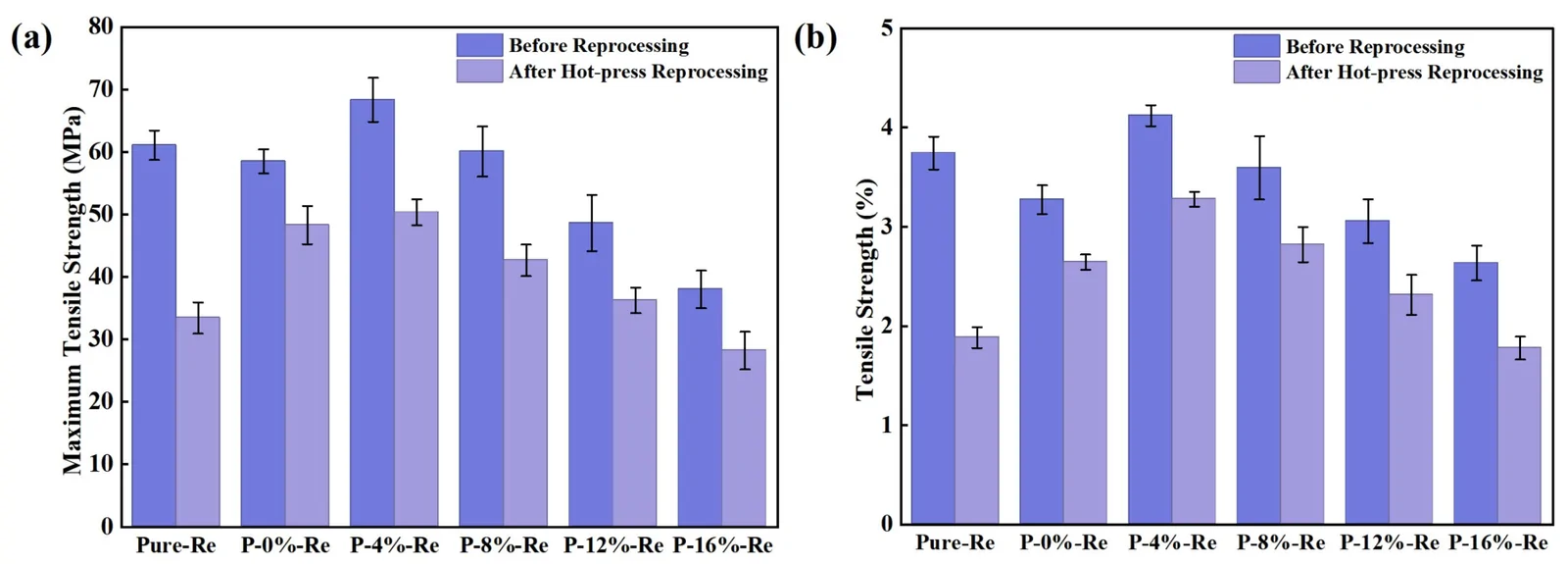
Mechanical properties of the epoxy resin composites before and after hot-press reprocessing at 180 °C and 15 MPa for 4 h: (**a**) maximum tensile strength and (**b**) elongation at break. Tensile testing was performed according to ASTM D638 at room temperature.

Figure 8 shows the stress–strain curves of composites with different PBO@SiO_2_ fiber contents before and after reprocessing. As shown in Figure 8a, before reprocessing, the tensile strengths of P-0%, P-4%, P-8%, P-12%, and P-16% are 58.25, 67.97, 60.18, 48.88, and 38.14 MPa, respectively, and their elongations at break are 3.29%, 4.20%, 3.65%, 3.08%, and 2.68%, respectively. The tensile strength and elongation at break first increase and then decrease with increasing PBO@SiO_2_ fiber content, reaching maximum values at a fiber content of 4 wt%.

The tensile strength and elongation at break reach their maximum values at 4 wt% PBO@SiO_2_ and then decrease with further fiber addition. SEM, EDS, and XPS confirm that the treatment changes the fiber surface morphology and chemistry, while Figure 3d–g confirms local contact between the coated fibers and matrix. The uncoated-fiber controls in Figure S7 further show generally higher tensile and flexural strengths for PBO@SiO_2_/EP than for PBO/EP at matched contents, which is consistent with a beneficial coating-related interphase effect. The non-monotonic composition dependence may reflect a balance between reinforcement and the increasing influence of dispersion heterogeneity, porosity, and fiber/matrix interfacial density at higher short-fiber contents.

As shown in Figure 8b, after reprocessing, the tensile strengths of P-0%-Re, P-4%-Re, P-8%-Re, P-12%-Re, and P-16%-Re are 48.83, 51.09, 43.10, 36.70, and 28.62 MPa, respectively, and their elongations at break are 2.65%, 3.28%, 2.81%, 2.34%, and 1.78%, respectively. Both tensile strength and elongation at break decrease after reprocessing. For the fiber-free P-0% sample, the tensile strength decreases from 58.25 to 48.83 MPa, while the elongation at break decreases from 3.29% to 2.65%. These results demonstrate that the reduction in mechanical properties also occurs in the absence of PBO@SiO_2_ fibers. The SEM images show more visible cracks in Pure-Re (Figure S1b), whereas P-0%-Re exhibits a denser fracture surface with fewer cracks (Figure 3i), demonstrating differences in the reconstructed fracture morphology of the two epoxy networks. During reprocessing, the original cured materials were converted into discrete particles by grinding and subsequently consolidated by hot pressing. Thermally activated dynamic-bond exchange promotes network rearrangement and particle-interface reconstruction during hot pressing, contributing to the observed retention of mechanical properties.

### 3.7. Thermal Conduction Mechanism of PBO@SiO_2_/EP Composites

**Figure 9:**
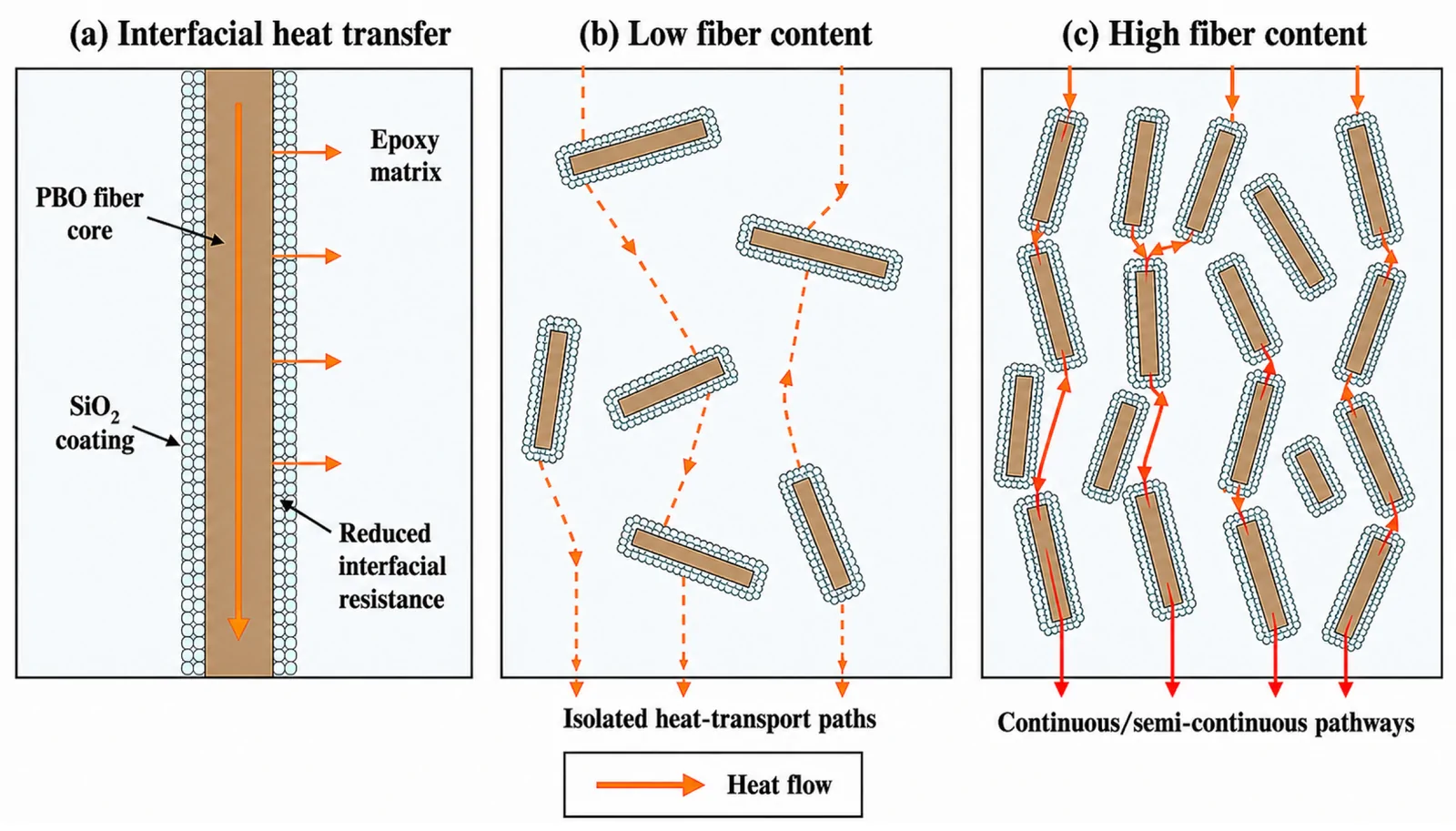
Schematic illustration of the thermal conduction mechanism in PBO@SiO_2_/EP composites.

Figure 9 shows the thermal conduction mechanism of the PBO@SiO_2_/EP composites. For the P-0% sample without PBO@SiO_2_ fibers, heat is mainly transferred through molecular chain vibrations in the epoxy resin. Because epoxy resin is a typical amorphous polymer with disordered molecular chains, phonons are easily scattered during transport, resulting in low thermal conductivity.

After PBO@SiO_2_ fibers are introduced, the measured thermal-conductivity trend is consistent with fiber-assisted heat transport. At low contents, individual fibers and their surrounding interphases contribute mainly as isolated transport elements; as the content increases, reduced interfiber spacing can increase the probability of near-contact or contact and promote cooperative pathways. Infrared thermography after identical preheating reveals a distinct transient thermal response after fiber incorporation (Figure S5). Together with the measured thermal-conductivity results and finite-element temperature and heat-flux fields in Figure S6, these observations support fiber-assisted heat transport and redistribution of heat flux through the fibrous phase. The finite-element fields in Figure S6 and the coated/uncoated comparison in Figure S7 are likewise consistent with redistribution of heat flux through the fibrous phase and a contribution from the modified interphase. Collectively, these observations support the proposed mechanism.

### 3.8. Chemical Degradation of PBO@SiO_2_/EP Composites

**Figure 10:**
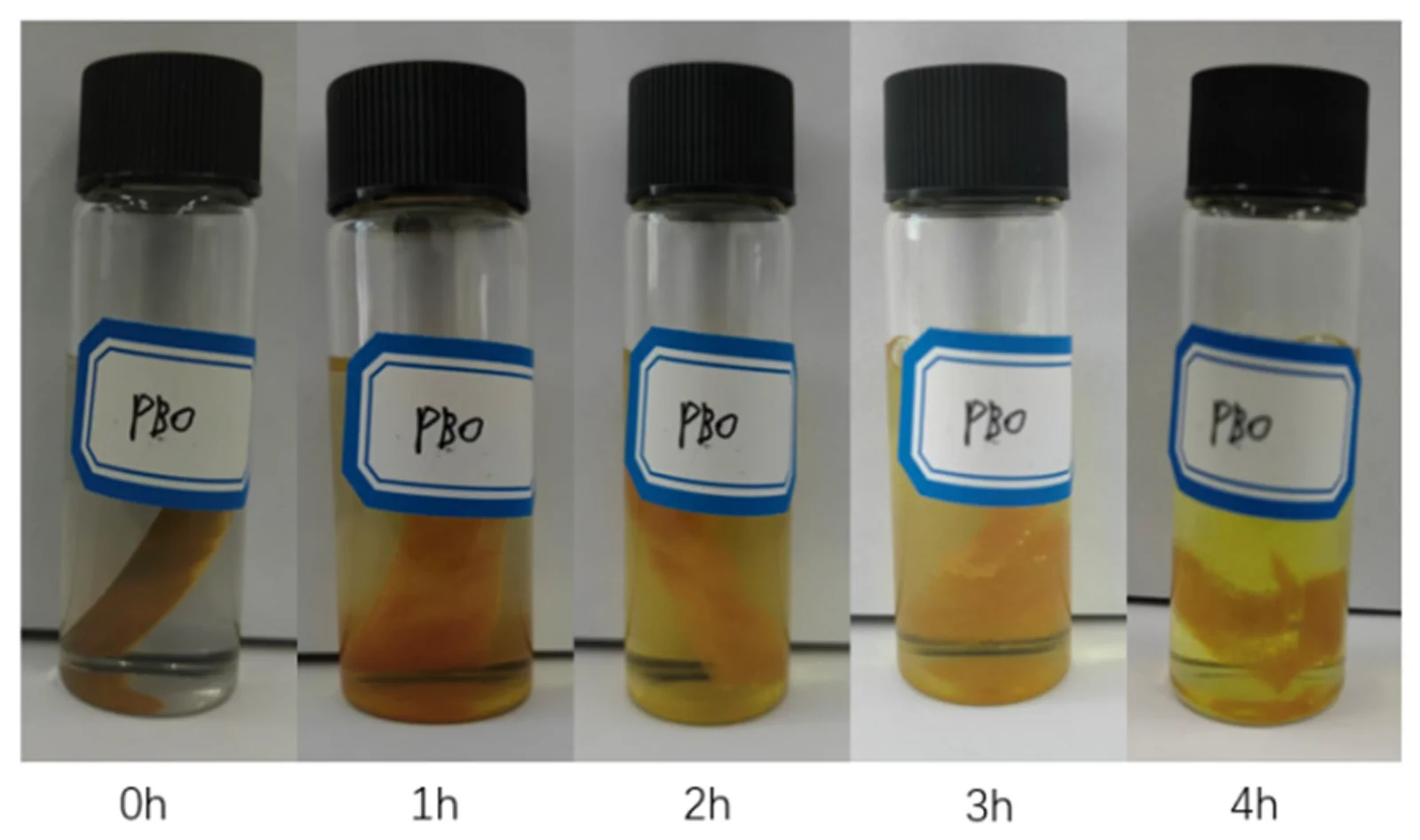
Digital photographs showing the n-hexylamine-induced degradation process of the P-12% composite at 90 °C for 0–4 h.

To evaluate the chemical degradability of PBO@SiO_2_/EP composites and the recoverability of reinforcing fibers, the P-12% sample was immersed in n-hexylamine at 90 °C. The degradation behavior was qualitatively evaluated by recording the macroscopic changes in the sample at different immersion times (Figure 10). The results show that P-12% was completely degraded within 4 h. During degradation, the resin matrix gradually dissolved, while the residual PBO@SiO_2_ fibers maintained their original morphology without obvious corrosion or dissolution.

The rapid degradation of P-12% is mainly attributed to the dynamic reversibility of imine bonds in the resin network. As a primary amine, n-hexylamine can react with imine bonds in the network through imine exchange, gradually depolymerizing the cross-linked network into soluble oligomers or small-molecule fragments. As the network is disrupted, the resin matrix detaches from the PBO@SiO_2_ fiber surface, enabling effective separation between the resin and the reinforcing fibers. In this qualitative assessment, complete degradation refers to the disappearance of the intact bulk specimen and the macroscopic separation of the resin matrix from the reinforcing fibers under the specified experimental conditions.

## 4. Conclusions

In this work, a recyclable epoxy composite with improved thermal conductivity was developed by combining a network containing imine and disulfide linkages with SiO_2_-coated PBO fibers. The vanillin-based VOEP component introduces imine linkages, while 2-AFD introduces disulfide linkages. Their coexistence provides two potential dynamic pathways for thermally activated rearrangement and amine-assisted degradation. The coexistence of imine and disulfide linkages provides complementary pathways for thermally activated network rearrangement and amine-induced degradation. DSC and DMA further show substantial retention of the thermal transition and dynamic-mechanical response after reprocessing, supporting the role of the combined dynamic-bond design in network reconstruction.

The sol–gel treatment produced a silica-rich surface layer on PBO fibers, as supported jointly by SEM/EDS and XPS. The treated fibers exhibit a rougher surface, higher surface O and Si contents, and increased O/C and Si/C ratios. The coated/uncoated controls in Figure S7 show generally improved thermal conductivity and mechanical strength for PBO@SiO_2_/EP at matched fiber contents.

The incorporation of PBO@SiO_2_ fibers effectively improved the thermal conductivity of the recyclable epoxy matrix. The thermal conductivity increased from 0.222 W/(m·K) for P-0% to 0.267 W/(m·K) for P-12%, representing a 20.3% enhancement. Although P-16% showed a higher thermal conductivity, its breakdown strength and mechanical properties decreased more significantly. Therefore, P-12% provided a better balance among thermal conductivity, dielectric performance, and mechanical properties. At 50 Hz, P-12% exhibited a dielectric constant of 4.26, a dielectric loss of 0.006, and a breakdown strength of 44.19 kV/mm.

PBO@SiO_2_ fibers also improve the thermal stability of the recyclable epoxy composites. Compared with P-0%, the T30%, Tmax, and THRI values of P-12% increase by 13.61, 6.13, and 4.04 °C, respectively. After hot-press reprocessing, P-12%-Re retains 92.1% of the original thermal conductivity and 83.8% of the original breakdown strength. DSC and DMA further show that the thermal transition and dynamic-mechanical response are substantially retained after reprocessing. These findings support thermally activated network rearrangement arising from the combined dynamic-bond design.

The P-12% composite undergoes macroscopic matrix disintegration in n-hexylamine at 90 °C within 4 h, and the recovered PBO@SiO_2_ fibers retain their overall morphology and Si distribution. A representative ester-only single-dynamic epoxy system is included in Figure S7 as a benchmark for initial electrothermal and mechanical properties under matched test conditions. Overall, the VOEP/DGEBA/2-AFD network and PBO@SiO_2_ surface engineering provide a practical route toward recyclable insulating epoxy composites with balanced thermal, dielectric, mechanical, and fiber-recovery performance.

## Data Availability

The raw data supporting the conclusions of this article will be made available by the authors on request.

## Supplementary Materials

The following supporting information can be downloaded at: https://www.mdpi.com/article/10.3390/polym18172118/s1, Figure S1: SEM images and EDS elemental mapping of the epoxy matrices and recovered fibers: (a) fracture surface of Pure before reprocessing; (b) fracture surface of Pure-Re after hot-press reprocessing; (c) PBO@SiO_2_ fibers recovered from the P-12% composite after n-hexylamine-induced degradation at 90 °C for 4 h; and (d) corresponding Si elemental mapping of the recovered fibers; Figure S2: XPS survey spectra and high-resolution Si 2p spectra of pristine PBO and PBO@SiO_2_; Table S1: Surface elemental compositions obtained from XPS; Figure S3: DSC curves of P-0%, P-12%, and P-12%-Re; Figure S4: DMA results of P-0%, P-12%, and P-12%-Re: (a) storage modulus (E′) as a function of temperature; (b) Tanδ as a function of temperature; Figure S5: Infrared cooling responses and representative thermal images of P-0% and P-12% after identical preheating; Figure S6: Finite-element model and simulated temperature and heat-flux fields under identical thermal boundary conditions; Figure S7: Electrical, thermal, dielectric, and mechanical properties of PBO/EP and PBO@SiO_2_/EP composites prepared with a representative ester-containing EP/glutaric-anhydride epoxy control matrix at different fiber contents: (a) Weibull plots of electrical breakdown strength; (b) thermal conductivity; (c) frequency-dependent dielectric constant; (d) frequency-dependent dielectric loss; (e) tensile strength; and (f) flexural strength.
