# Supplementary material for: Study of Dielectric and Thermal Conductivity Properties of Recyclable Epoxy Resin Reinforced with SiO2-Coated Poly(p-phenylene benzobisoxazole) Fibers
Source: Polymers (Basel). 2026 Aug 31;18(17):2118. doi: 10.3390/polym18172118 (PMC13568119; doi:10.3390/polym18172118)
Supplement: Supplementary file 1 [file polymers-18-02118-s001.zip › polymers-4439669-supplementary.pdf]

Study of Dielectric and Thermal Conductivity Properties of Recyclable Epoxy Resin Reinforced with SiO_2-Coated Poly(p-phenylene benzobisoxazole) Fibers

Xiangyu Zhao *, Difei Wang, Xueting Wang, Xiaohui He

Electric Power Research Institute, State Grid Heilongjiang Electric Power Company Limited, Harbin 150030, China
* Correspondence: 18846140520@163.com

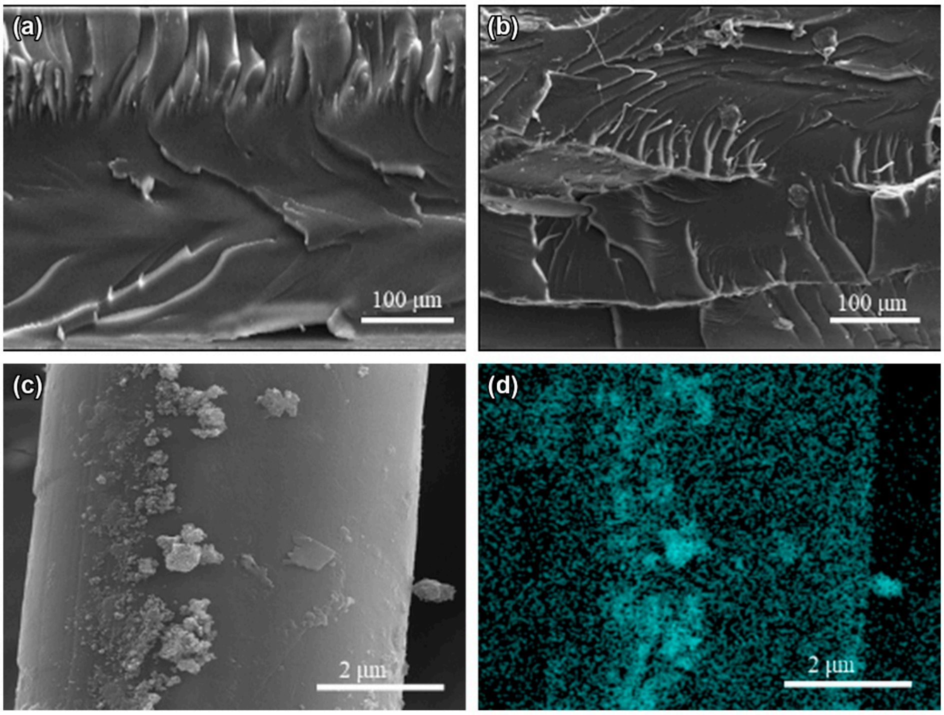

Figure S1. SEM images and EDS elemental mapping of the epoxy matrices and recovered fibers: (a) fracture surface of Pure before reprocessing; (b) fracture surface of Pure-Re after hot-press reprocessing; (c) PBO@SiO_2 fibers recovered from the P-12% composite after n-hexylamine-induced degradation at 90 °C for 4 h; and (d) corresponding Si elemental mapping of the recovered fibers.

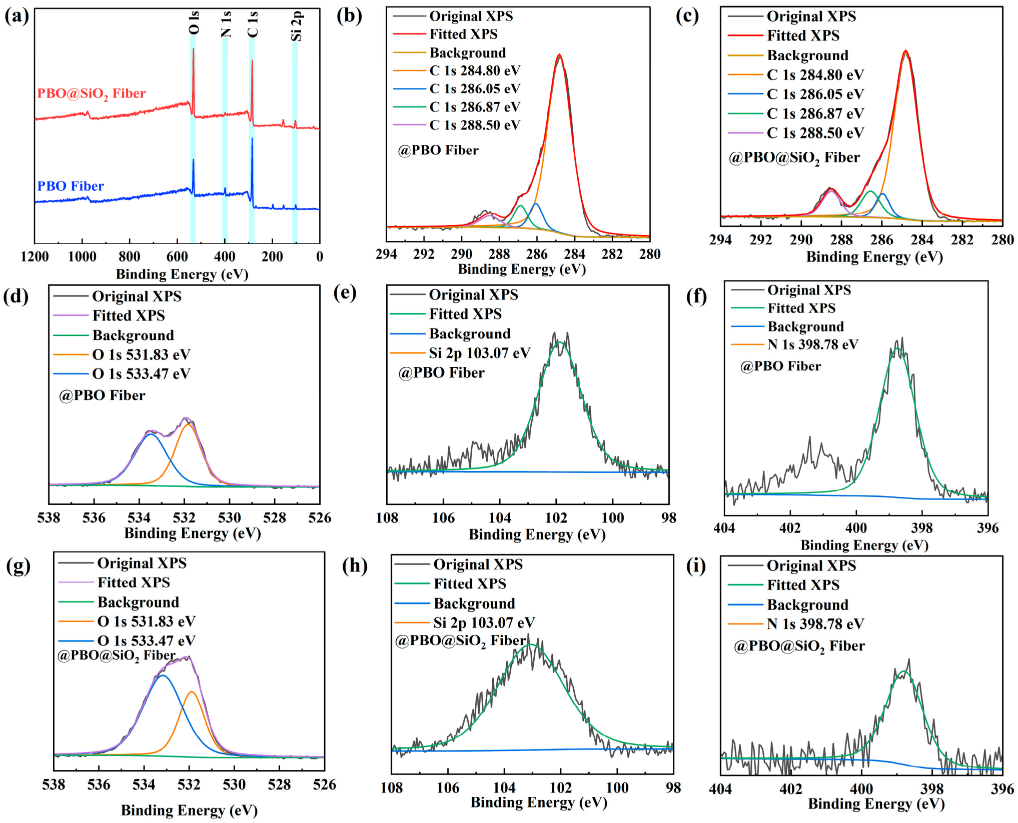

Figure S2. XPS survey spectra and high-resolution Si 2p spectra of pristine PBO and PBO@SiO_2.

Table S1. Surface elemental compositions obtained from XPS.

Sample	C (at.%)	N (at.%)	O (at.%)	Si (at.%)	Si/C	O/C	Si 2p BE (eV)
PBO	74.49	5.17	17.02	3.32	0.045	0.228	102.15
PBO@SiO_2	67.07	1.91	25.21	5.80	0.086	0.376	103.15

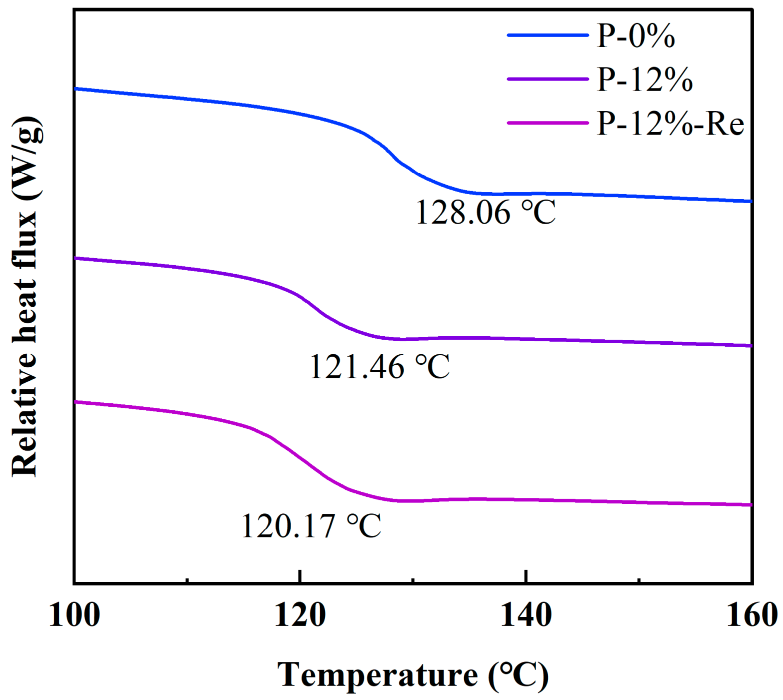

Figure S3. DSC curves of P-0%, P-12%, and P-12%-Re.

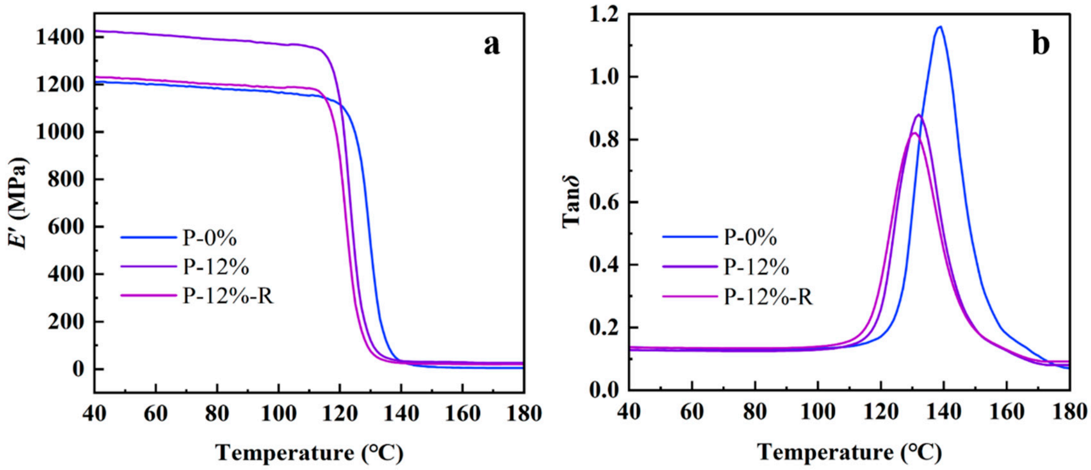

Figure S4. DMA results of P-0%, P-12%, and P-12%-Re: (a) storage modulus (E') as a function of temperature; (b) Tanδ as a function of temperature

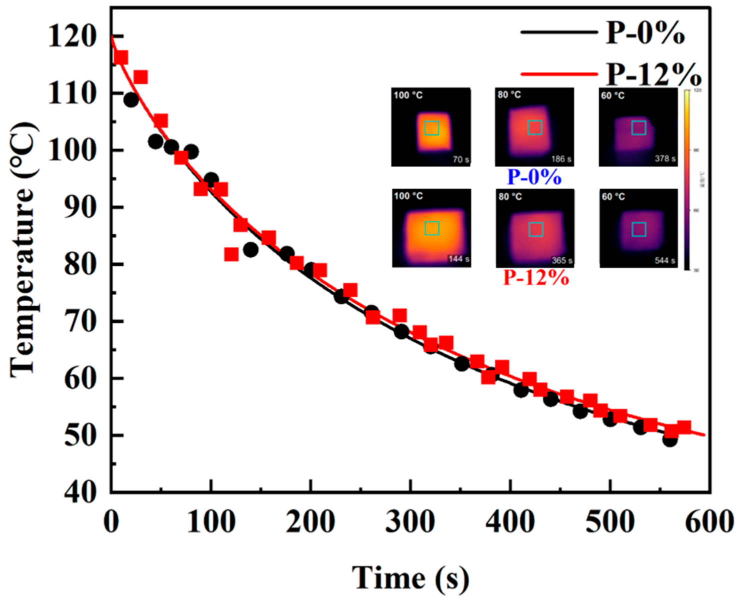

Figure S5. Infrared cooling responses and representative thermal images of P-0% and P-12% after identical preheating.

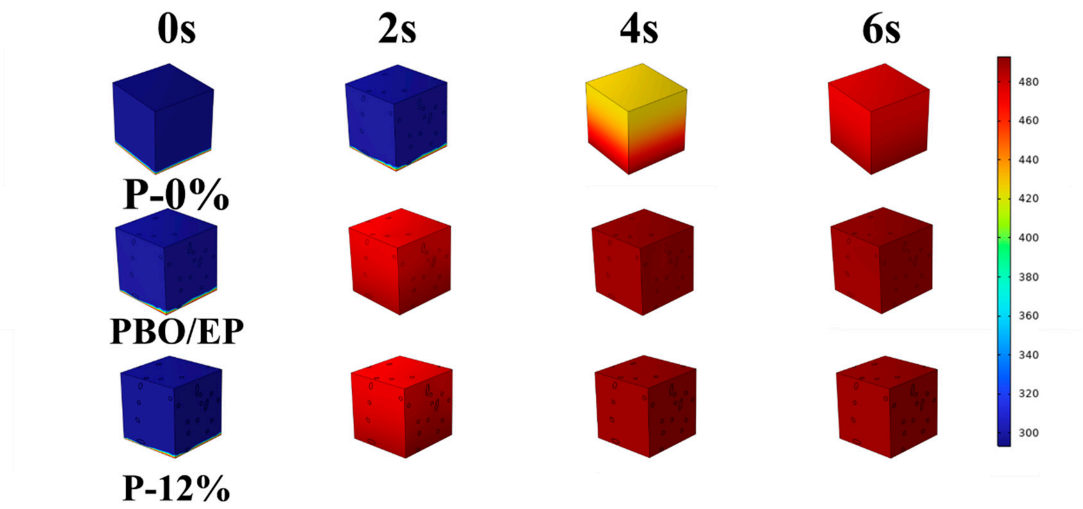

Figure S6. Finite-element model and simulated temperature and heat-flux fields under identical thermal boundary conditions.

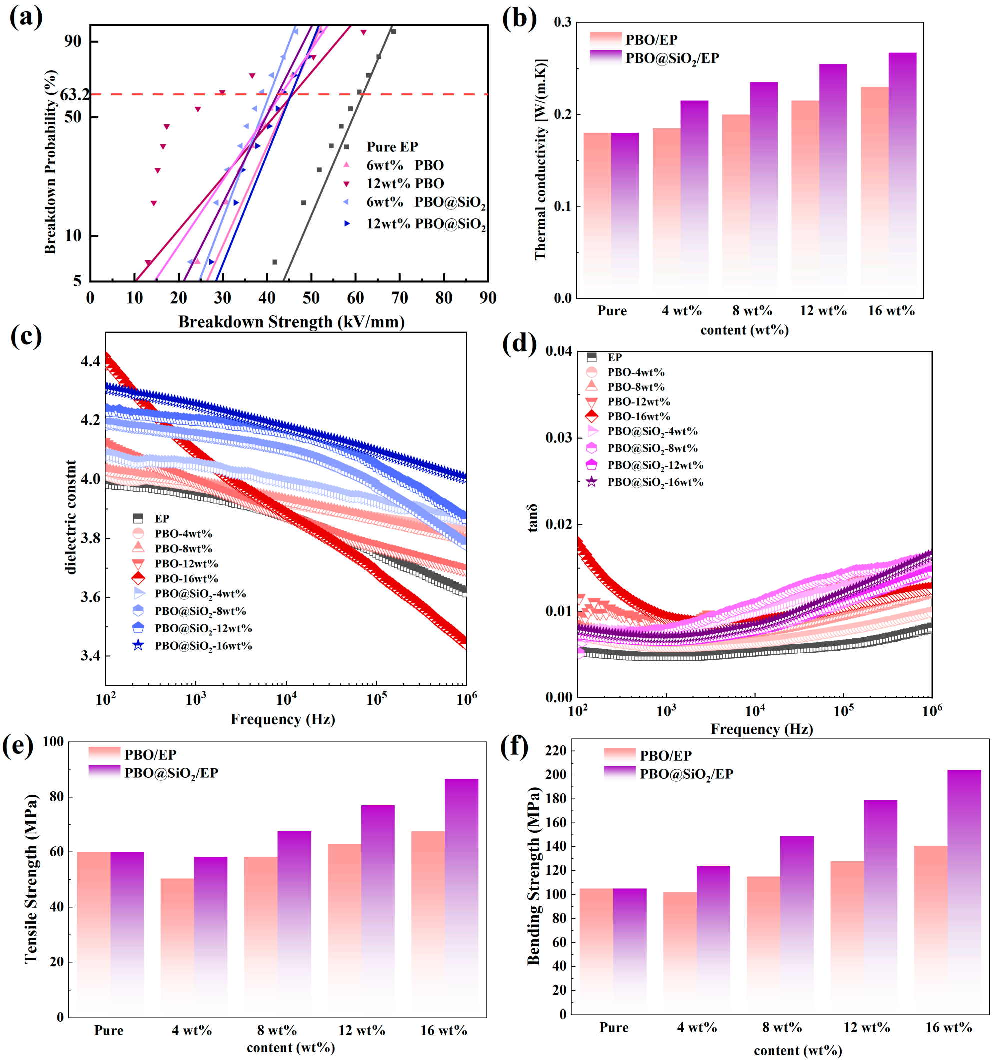

Figure S7. Electrical, thermal, dielectric, and mechanical properties of PBO/EP and PBO@SiO_2/EP composites prepared with a representative ester-containing EP/glutaric-anhydride epoxy control matrix at different fiber contents: (a) Weibull plots of electrical breakdown strength; (b) thermal conductivity; (c) frequency-dependent dielectric constant; (d) frequency-dependent dielectric loss; (e) tensile strength; and (f) flexural strength.

The ester-containing control composites were prepared from epoxy resin and glutaric anhydride using a stoichiometric formulation. The same types of PBO and PBO@SiO_2 fibers were incorporated at 0, 4, 8, 12, and 16 wt%, and the electrical, thermal, dielectric, tensile, and flexural properties were measured under the same corresponding test conditions used for the main composite series. Figure S7 provides supplementary evidence for coating-related electrothermal and mechanical property trends.
